# circ_SMAD2 regulate colorectal cancer cells proliferation through targeting miR-1258/RPN2 signaling pathway

**DOI:** 10.7150/jca.50888

**Published:** 2021-01-16

**Authors:** Wei Zhang, Gang Wu, Peichun Sun, Yuanzeng Zhu, Han Zhang

**Affiliations:** Department of Gastrointestinal Surgery, Henan Provincial People's Hospital, People's Hospital of Zhengzhou University, School of Clinical Medicine, Henan University, Zhengzhou, Henan 450003, People's Republic of China.

**Keywords:** circ_SMAD2, colorectal cancer, miR-1258, RPN2

## Abstract

Circular RNAs (circRNAs) are associated with various diseases, including cancers. However, their roles in colorectal cancer (CRC) have not been established. Hsa_circ_0000847 (circ_SMAD2) is a novel circRNA that was found to be elevated in CRC cell lines and tissues. High circ_SMAD2 levels were positively correlated with CRC clinicopathological features. Functional assays revealed that circ_SMAD2 enhanced CRC cell invasion, proliferation, and tumor growth. Mechanistically, circ_SMAD2 elevated Ribophorin II (RPN2) levels by inhibiting miR-1258. Therefore, circ_SMAD2 is a potential indicator for CRC progression.

## Introduction

Globally, colorectal cancer (CRC) is the 3^rd^ most common malignancy. It is highly associated with cancer related mortalities 1, 2. Therapeutic options for CRC include surgical resection, radiotherapy, chemotherapy, and vital adjuvant treatment. These options significantly improve survival outcomes 3, 4. However, the 5-year survival of patients with distal metastasis is low (12.5%) 5. To identify novel biomarkers and therapeutic targets for CRC, more studies aimed at elucidating its molecular basis are needed.

Circular RNAs (circRNAs) modulate multiple biological processes in CRC 6, 7. Ge et al reported that down-regulation of circMTO1 suppresses the proliferative and invasive abilities of CRC through the Wnt/beta-catenin signaling pathway 8. In addition, circITGA7 inhibits CRC cell proliferation and metastasis by suppressing the Ras signaling pathway while enhancing ITGA7 expression 9. Elevated hsa_circ_0136666 levels promote the invasion and proliferation of CRC by molecularly sponging miR‐136 10. However, the roles of hsa_circ_0000847 (circ_SMAD2) in the progression of CRC have not been established. circ_SMAD2 have been shown to be elevated in CRC, therefore, we hypothesized that circ_SMAD2 may be oncogenic.

miRNAs promote CRC development and progression 11. Li et al documented that miR-205 suppresses CRC metastasis by targeting CREB1 12. The down-regulation of miR-143 in CRC has been shown to suppress cell progression by regulating the expression of MMP7 13. miR-1258 is significantly down-regulated in CRC 14, 15. However, the relationships between circ_SMAD2 and miR-1258 have not been determined.

This study aimed at determining the roles of circ_SMAD2 in CRC progression and metastasis, as well as its mechanism of action.

## Materials and Methods

### Clinical specimens

This study was approved by the ethical committee of Henan Provincial People's Hospital. The participants who agreed to be enrolled in this study were required to sign an informed consent. A total of 53 pairs of CRC tissue and adjacent normal tissue (ANT) specimens were collected between 2015 and 2016. Before the surgical procedures, none of the study participants had undergone radiotherapy, chemotherapy, or targeted therapy. After surgical resection, the specimens were immediately frozen in liquid nitrogen.

### Cell culture and transfection

Human colonic mucosal epithelial cell line (NCM460) and CRC cell lines (HT-29, HCT116, LoVo, SW480 and SW620) were acquired from American type culture collection (ATCC). Cells were grown in Dulbecco's modified Eagle's medium (DMEM, Sigma) supplemented with 10% fetal bovine serum (FBS, Gibco), 100 µg/mL streptomycin and 100 µg/mL penicillin at 37 °C, in a humified incubator with 5% CO_2_.

Anti circ_SMAD2 siRNA, miR-1258 mimics, pcDNA3.1/RPN2 and corresponding controls were offered by Shanghai GenePharma Co., Ltd. The Lipofectamine 3000 reagent (Invitrogen) was added to facilitate transfection as per the manufacturer's instructions.

### qRT-PCR

miRNeasy Mini Kit (Qiagen) was employed for RNA isolation. After treatment with DNase I (Sigma), total RNA was quantified at the ratio of A260/A280. Total RNA was reversely transcribed into cDNA using SuperScript VILO reagent kit (Invitrogen) or miScripIIRT kit (Qiagen). The synthesized cDNA was used for qRT-PCR with the SYBR Green PCR Master Mix (Vazyme, Nanjing, China). The relative expression was analyzed by the 2^-ΔΔCt^ method. Primers: circ_SMAD2-F: 5'-TATTCCAGAAACGCCACCTCC-3', circ_SMAD2-R: 5'- GCAAGCCACGCTAGGAAAAC-3', GAPDH or U6 served was used as an internal.

### CCK-8 assay

Cells were seeded in 96-well plates (2×10^3^ cells/well) and allowed to adhere. 10μL of Cell Counting Kit-8 solution (CCK-8, Dojindo, Japan) were added into each well and cultured for 2 h. Optical density was recorded at 450nm by a microplate reader. Proliferation rates were measured at 24, 48, 72 and 96 h after transfection.

### Flow cytometry assay

The transfected cells were dissociated with trypsin and twice rinsed with PBS 1X. Cells were then fixed in 70% ethanol at 4 °C overnight. Cell cycle analyses were done using a FACS Calibur flow cytometer (BD Biosciences) after propidium iodide (PI) staining for 30 min in the presence of RNase A (KeyGEN Biotech, Nanjing, China).

### Colony formation assay

After transfection, cells were cultured in 6-well plates for 2 weeks in media containing 10% FBS. They were fixed in methanol and stained with 0.5% crystal violet. The number of colonies formed were then counted.

### Transwell invasion assay

Cell invasion was explored using the Transwell chambers with Matrigel (Millipore, Billerica, MA, USA). Briefly, the upper chambers of Transwell inserts were covered with 1×10^5^ transfected cells resuspended in 200 μL FBS-free DMEM medium. A volume of 500 μL DMEM+10% serum was added to the lower chamber. After 48 h, cells seeded in the upper and lower chambers were obtained using cotton swabs, fixed in 4% PFA and stained with 0.1% crystal violet (Beyotime, China) for 30 min. Stained cells were counted using an upright microscope (Nikon, 200× magnification).

### Animal studies

BALB/c female nude mice, weight 16-20 g, aged 6-8 weeks, were bought from Laboratory Animal Co., Ltd. (Beijing Vital River; Beijing, China). 5×10^6^ transfected cells were subcutaneously injected into mice right flanks. During the experimental period, tumor volumes were calculated every 7 days by the equation: volume = 0.5 × length × width^2^. 35 days after inoculation, mice were euthanized. Mice experiments were approved by the ethics committee of Henan Provincial People's Hospital and we performed following the guidelines of the National Institute of Health.

### Dual-luciferase reporter assay

Circ_SMAD2 or RPN2 3'UTR with binding sites for wild-type or mutant miR-1258, were cloned into pmirGLO vector (Promega) and named WT-circ_SMAD2, MUT- circ_SMAD2, WT-RPN2 or MUT-RPN2. The corresponding luciferase reporter and miR-1258, or miR-NC, were co-transfected into the CRC cells. Luciferase activity was measured using a dual-luciferase reporter system (Gene, China).

### Actinomycin D, RNase R and Western blot assays

All these assays were performed as previously described 16, 17.

### Statistical analysis

All study data were analyzed using SPSS software (version 20.0) and presented as mean ± SD. Differences between experimental groups were analyzed Student's t-test or one-way ANOVA. *S*tatistical significance was set at p≤0.05.

## Results

### Circ_SMAD2 is significantly upregulated in CRC

**Figure 1:**
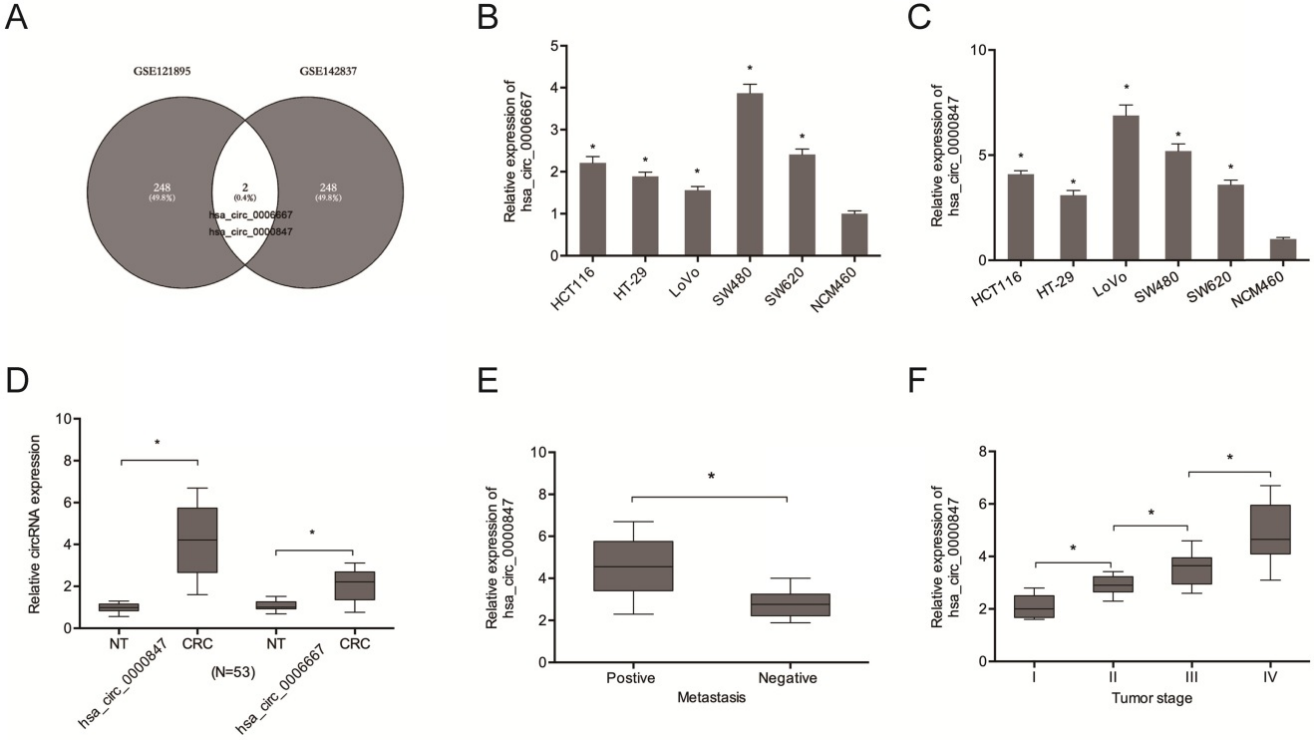
Hsa_circ_0000847 is upregulated in CRC. **(A)** Differentially expressed circRNAs in 2 circRNA microarray datasets (GSE121895 and GSE142837). **(B, C)** Relative hsa_circ_0000847 and hsa_circ_0006667 expression levels in CRC cell lines. **(D)** Relative hsa_circ_0000847 expression levels in CRC tissues. **(E, F)** High hsa_circ_0000847 expression level correlates with metastasis and advanced tumor stage in CRC patients. *p<0.05.

Analysis of circRNA expression in the CRC microarray datasets (GSE121895 and GSE142837) revealed an aberrant expression of Hsa_circ_0000847 and hsa_circ_0006667 (Figure 1A). Figure 1B-D shows the expression levels of hsa_circ_0000847 and hsa_circ_0006667 in CRC cell lines and tissues. Figure 1E-F shows that hsa_circ_0000847 was significantly upregulated in metastatic and advanced CRC.

**Figure 2:**
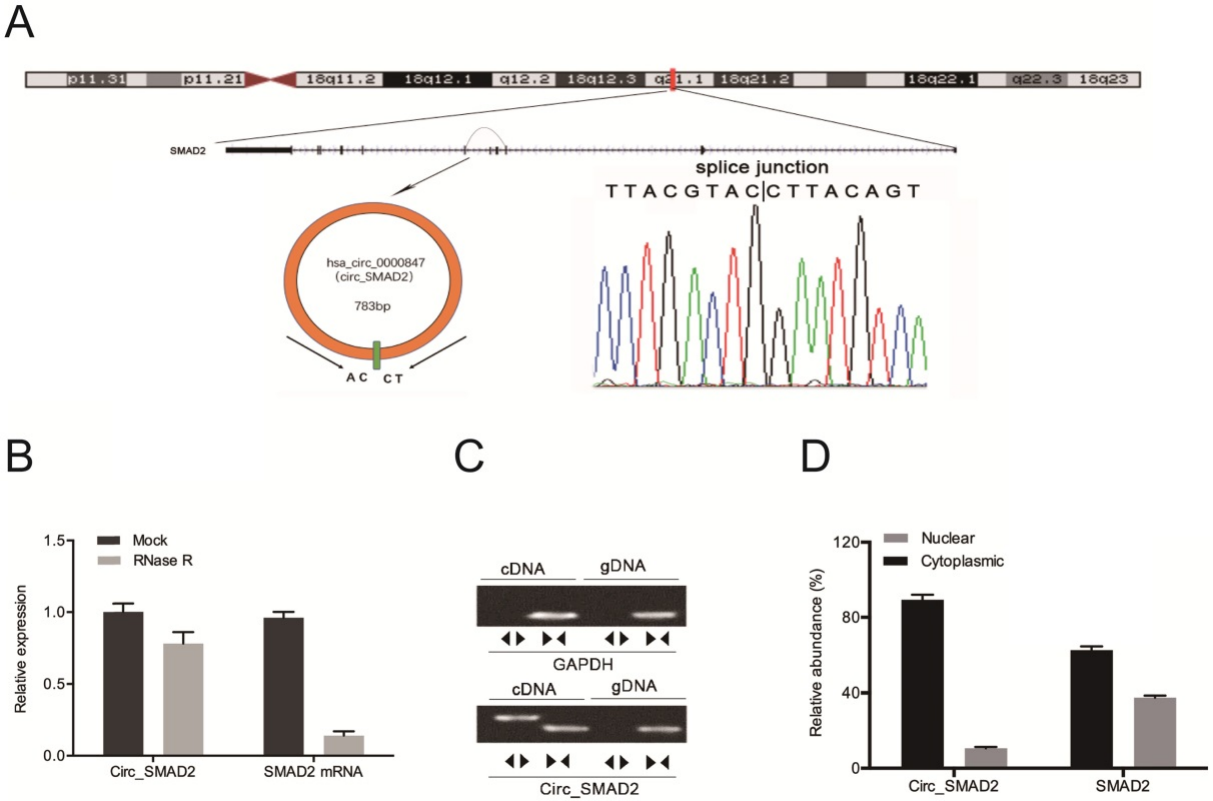
Identification of circ_SMAD2 in CRC and confirmation of its circular structure. **(A)** Schematic illustration of circ_SMAD2. **(B)** Circ_SMAD2 and SMAD2 mRNA levels were measured by RT‐qPCR in CRC cells treated with or without RNase R. **(C)** Divergent primers detected circ_SMAD2 in cDNA but not in gDNA. **(D)** Subcellular localization of circ_SMAD2 in CRC cells. *p<0.05.

Hsa_circ_0000847 is located on chromosome 18, and is comprised of exon 2-5 of its host gene, SMAD2 (Figure 2A). Circ_SMAD2 was found to be resistant to RNase R digestion (Figure 2B). The amplification of circ_SMAD2 with divergent primers revealed its amplification in cDNA, but not in gDNA (Figure 2C). Additionally, circ_SMAD2 was shown to be mainly localized in the cytoplasmic fraction. However, it was also present in the nuclear fraction (Figure 2D).

### Circ_SMAD2 knockdown inhibits CRC cell proliferation and invasion

**Figure 3:**
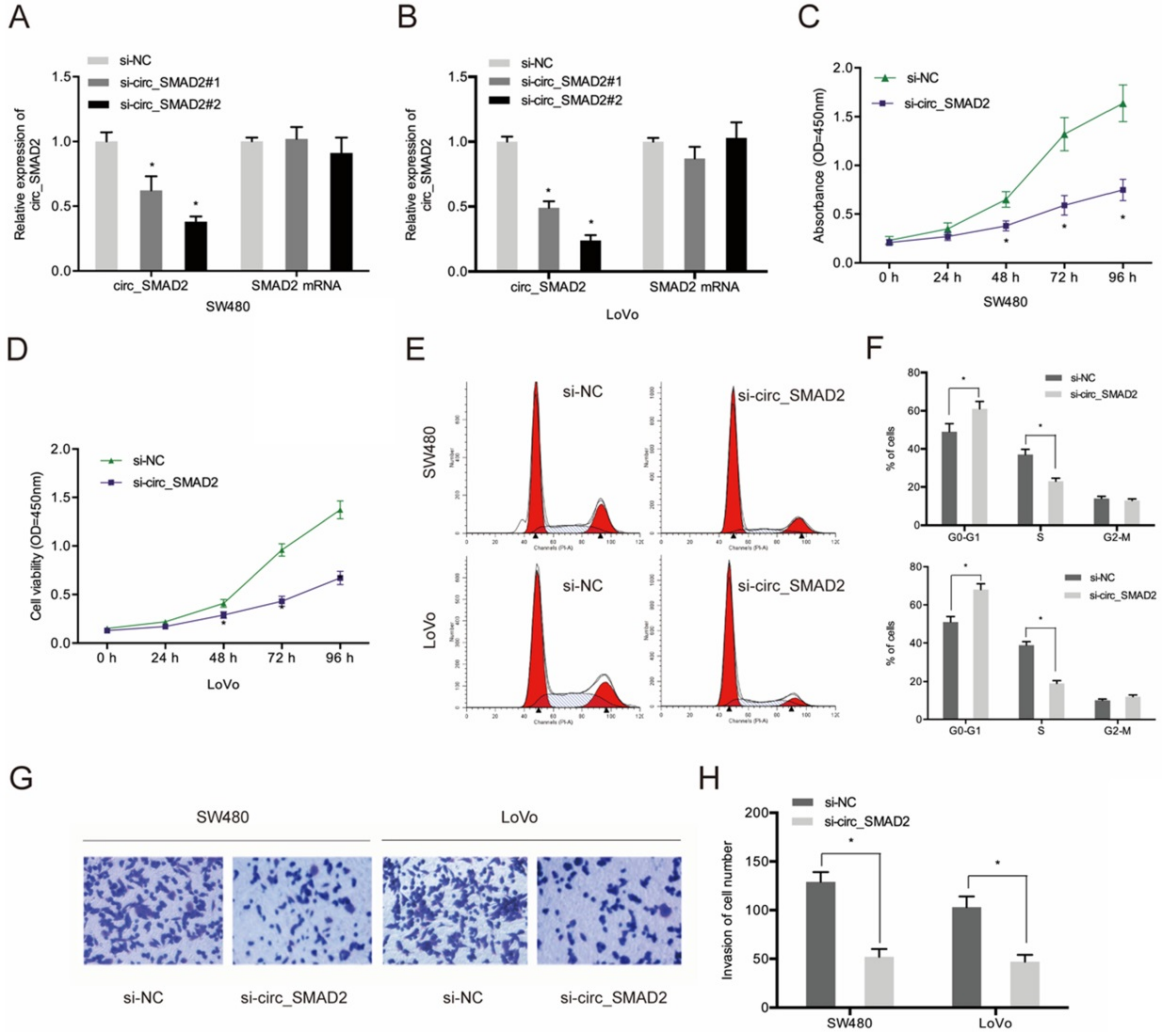
Suppressed circ_SMAD2 expression inhibits CRC cell proliferation, and invasion. **(A, B)** Si-circ_SMAD2 transfection efficiency was tested in CRC cells with si-NC as control. **(C, D)** CCK-8 assay evaluated the effect of si-circ_SMAD2 on cell proliferation. **(E, F)** Flow cytometry measured si-circ_SMAD2 effects on CRC cell cycle progression. **(G, H)** Transwell assays monitored the effect of si-circ_SMAD2 on CRC invasion. *p<0.05.

To determine the role of circ_SMAD2 in CRC, 2 siRNAs against its unique back splicing junction were used. The antisense spliced junction siRNAs significantly suppressed circ_SMAD2 expression without affecting linear SMAD2 mRNA levels (Figure 3A-B). The CCK-8 assay revealed that down-regulation of circ_SMAD2 suppressed the proliferation of SW480 and LoVo cells (Figure 3C-D). Figure 3E-F shows that upon circ_SMAD2 suppression, the cell cycle was arrested at G1/S phases in SW480 and LoVo cells (Figure 3E-F). In addition, circ_SMAD2 knockdown significantly suppressed *in vitro* CRC cell invasion (Figure 3G-H).

### Circ_SMAD2 directly interacts with miR-1258

**Figure 4:**
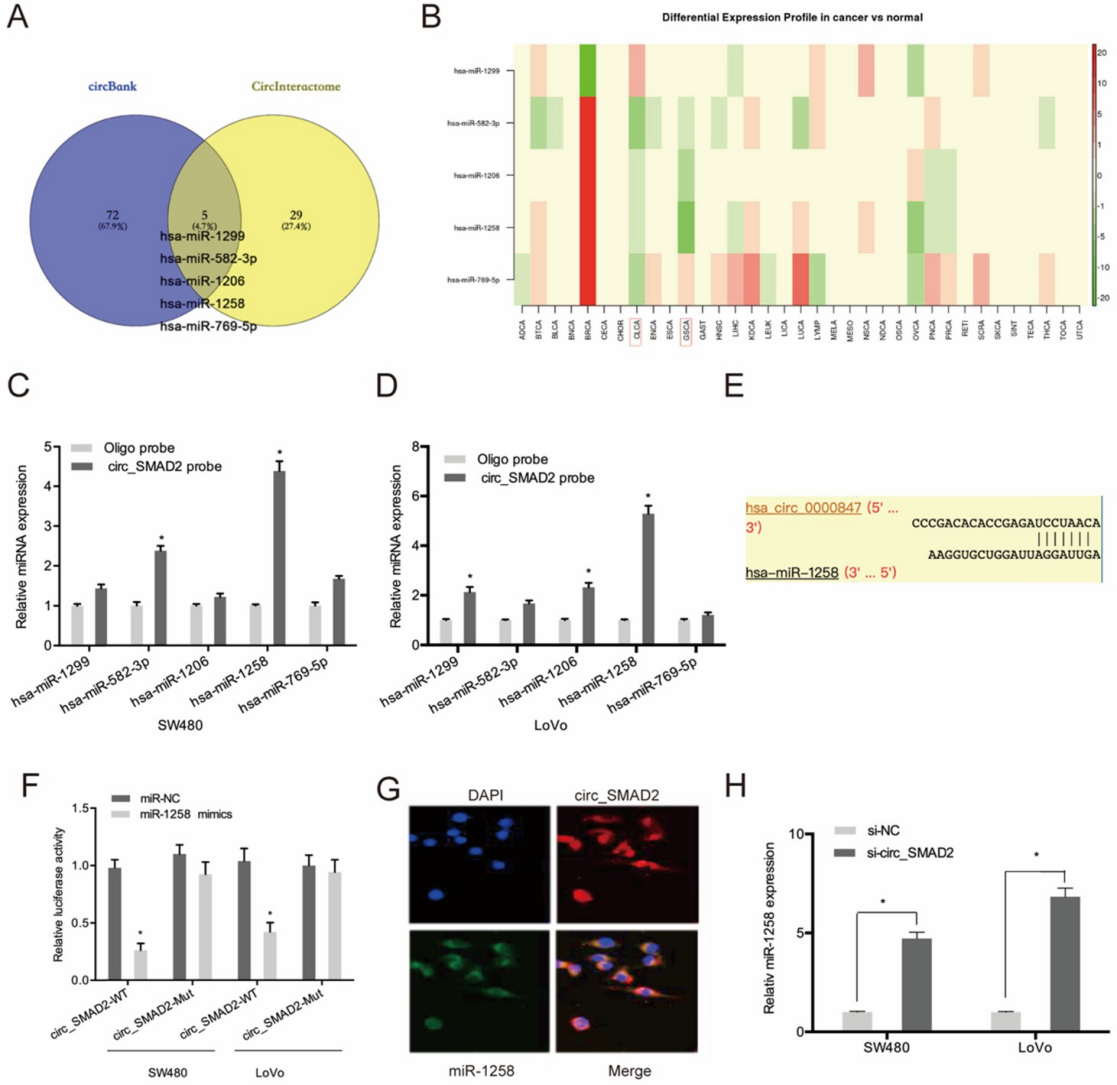
Circ_SMAD2 interacts with miR-1258. **(A)** The overlapping miRNAs from 2 online analysis tools (circbank and circInteractome). **(B)** The relative levels of 5 miRNA in TCGA database. **(C, D)** The relative levels of 5 miRNA candidates in the CRC cells lysates were detected by qRT-PCR. **(E, F)** Luciferase reporter assay was conducted to evaluate the interaction ability between circ_SMAD2 and miR-1258. **(G)** RNA FISH for circ_SMAD2 and miR-1258 was detected in CRC cells. **(H)** silencing of circ_SMAD2 increased the expression of miR-1258 in CRC cell lines. *p<0.05

To evaluate the mechanisms of circ_SMAD2 activity in CRC, circ_SMAD2 binding to miRNA was analyzed. Circbank and circInteractome analysis predicted 5 miRNAs (hsa-miR-1299, hsa-miR-582-3p, hsa-miR-1206, hsa-miR-1258, and hsa-miR-769-5p) that are potential circ_SMAD2 targets. The expression of these miRNAs was evaluated in the TCGA datasets (Figure 4A-B). The RNA pulldown analysis of whether these miRNAs physically interacted with circ_SMAD2 revealed the enrichment of miR-1258 in the circ_SMAD2 pull-down (Figure 4C-D). Figure 4E-F shows that miR-1258 significantly suppressed the luciferase activity of the WT-circ_0000847 reporter. RNA-FISH analysis confirmed the colocalization of circ_SMAD2/miR-1258 in the cytoplasm (Figure 4G). Furthermore, circ_SMAD2 silencing elevated miR-1258 expression in the CRC cell lines (Figure 4H).

**Figure 5:**
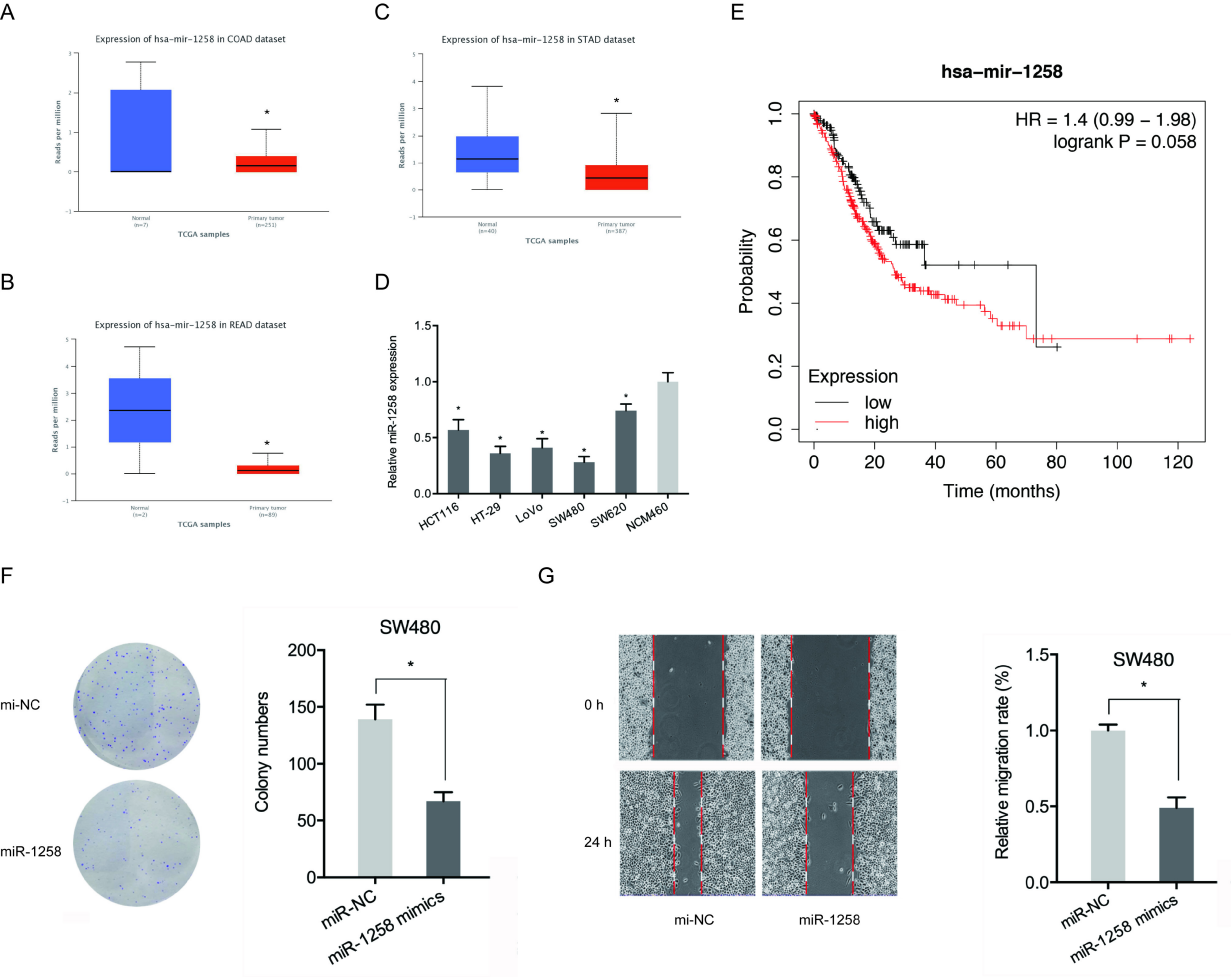
miR-1258 inhibits the proliferation and migration of CRC cells. **(A-C)** Expression of miR-1258 in COAD, READ and STAD tissues. **(D)** Expression of miR-1258 in CRC cell lines. **(E)** Kaplan-Meier analysis of overall survival based on miR-1258 expression. **(F, G)** Colony formation and wound healing assays showed that miR-1258 overexpression inhibited growth and migration of CRC cells * in vitro*. *p<0.05.

The roles of miR-1258 in CRC were also evaluated. Figure 5A-C shows that miR-1258 was significantly down-regulated in CRC tissues. qRT-PCR analysis revealed that miR-1258 levels were significantly low in CRC cell lines compared to normal cells (Figure 5D). Low miR-1258 levels were positively correlated with poor CRC prognosis (Figure 5E). Moreover, colony formation and wound healing assays confirmed that the over-expression of miR-1258 suppressed the *in vitro* migration and proliferation of CRC cells (Figure 5F-G). Therefore, circ_SMAD2 directly binds miR-1258 in CRC cells.

### RPN2 is a downstream target of miR-1258

**Figure 6:**
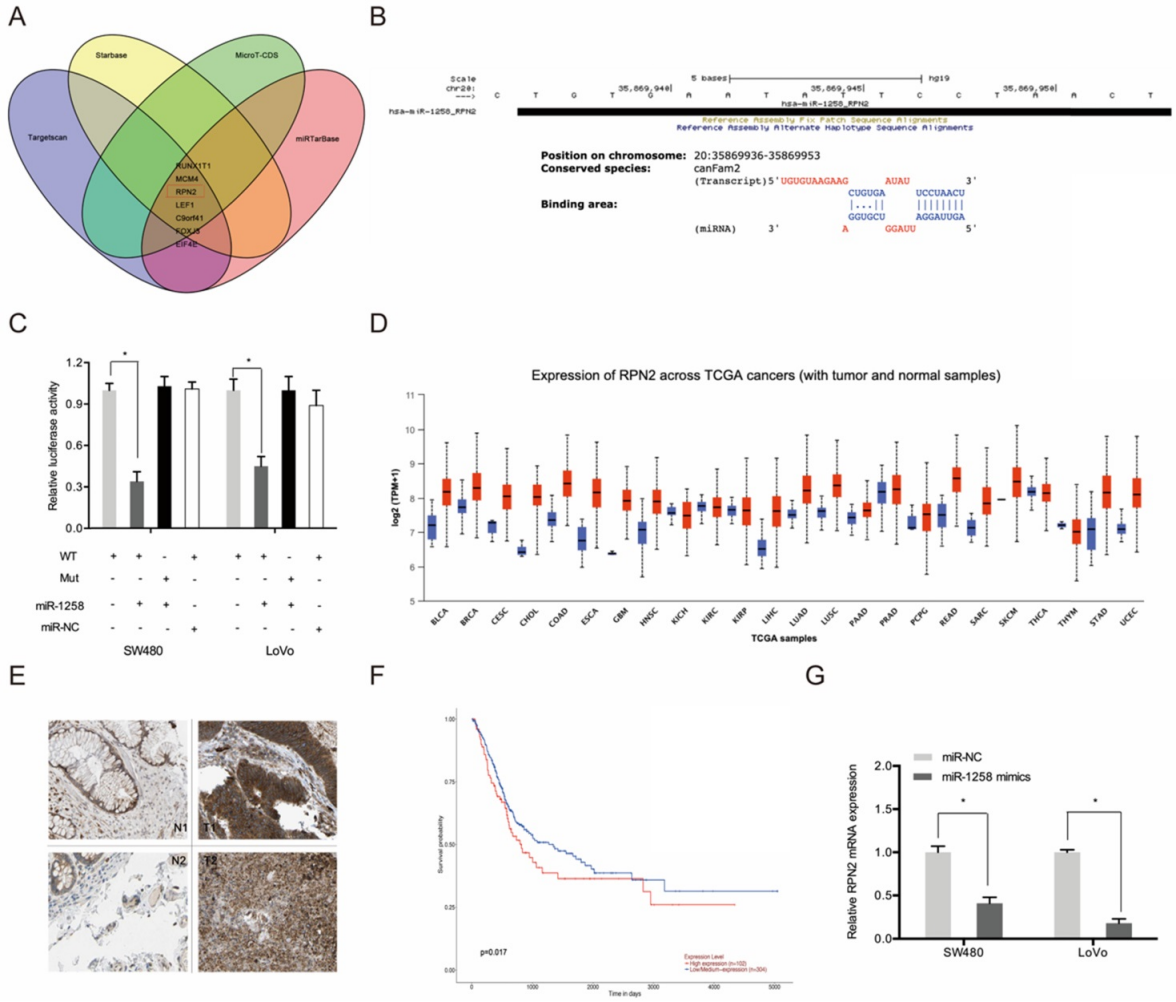
RPN2 is a downstream target gene of miR-1258 in CRC. **(A)** The overlapping miRNAs from 4 online analysis tools. **(B)** The binding site of miR-1258 to RPN2. **(C)** The relationship of miR-1258 and RPN2 was proved by luciferase reporter assay. **(D)** The RPN2 expression in TCGA dataset. **(E)** Relative expression of RPN2 in CRC tissues by IHC. **(F)** Kaplan-Meier analysis of overall survival based on RPN2 expression in CRC patients. **(G)** miR-1258 overexpression reduced RPN2 levels in CRC cells. *p<0.05.

Next, we used Targetscan, Starbase, miRTarBase and MircoT-CDS to predict miR-1258 targets and found that it could bind to RPN2 (Figure 6A-B). Luciferase reporter assays indicates that miR-1258 markedly suppressed luciferase activity of WT-RPN2 group (Figure 6C). TCGA dataset analysis revealed high RPN2 expression in most tumors, including CRC (Figure 6D). IHC analysis revealed high RPN2 levels in CRC tissues (T) relative to normal tissues (N) (Figure 6E). Kaplan-Meier analysis showed that CRC patients with high RPN2 levels exhibited poor overall survival (Figure 6F). Moreover, RT-qPCR analysis revealed that miR-1258 overexpression significantly reduced RPN2 levels in CRC cells (Figure 6G).

### circ_SMAD2 promotes CRC progression by regulating miR-1258/RPN2 axis

**Figure 7:**
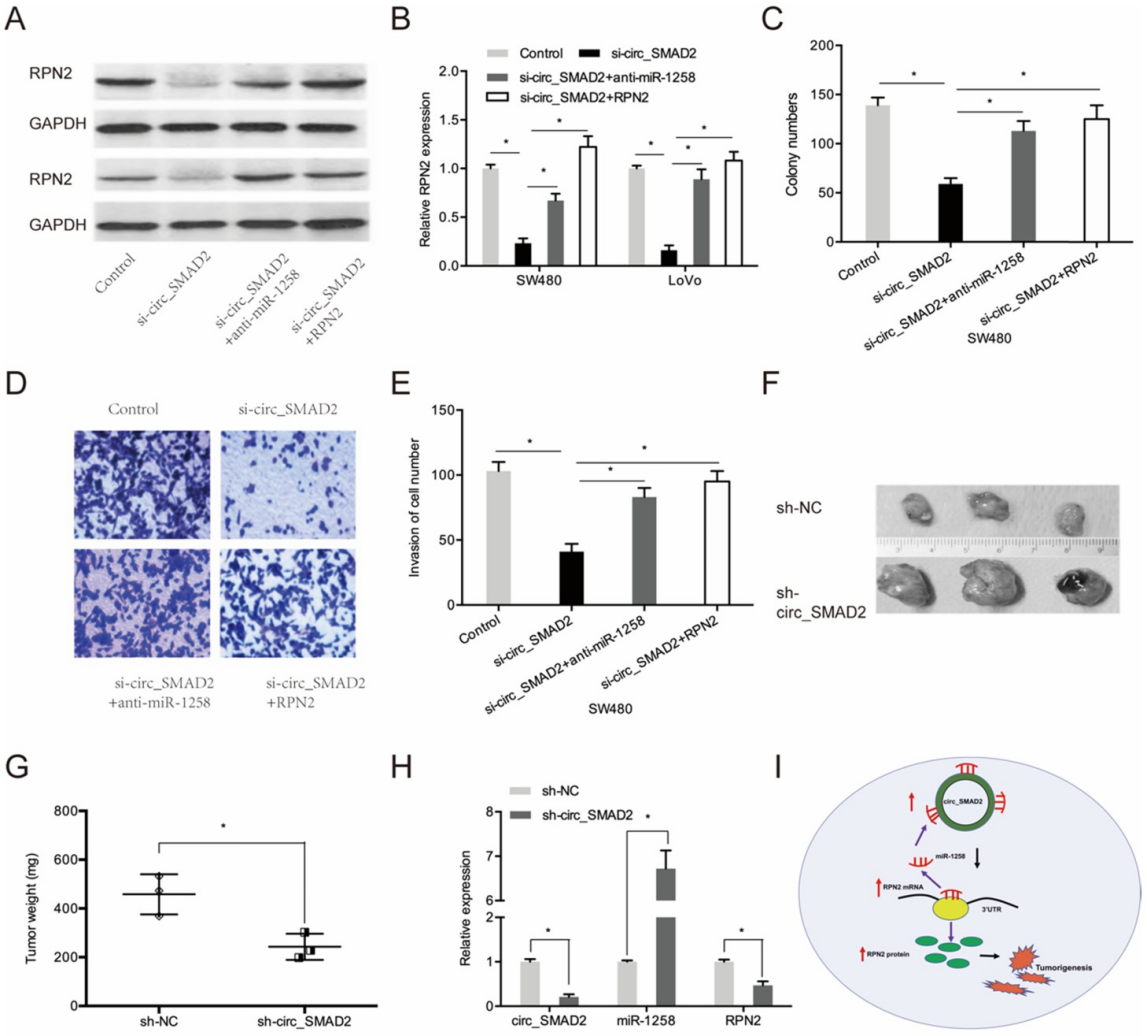
circ_SMAD2/miR-1258/RPN2 axis in CRC. **(A, B)** miR-1258 suppression (or RPN2 upregulation) abolished the effects of circ_SMAD2 suppression on RPN2 in CRC cells. **(C-E)** miR-1258 suppression (or RPN2 upregulation) abolished the effects of circ_SMAD2 suppression on the proliferation and invasion of CRC cells. **(F, G)** After 35 days, the mice were sacrificed, and the tumors were weighed. **(H)** The levels of circ_SMAD2, miR-1258 and RPN2 were detected by qRT-PCR. **(I)** The circ_SMAD2/miR-1258/RPN2 axis in CRC. *p<0.05.

Next, we investigated whether circ_SMAD2 promotes CRC proliferation and invasion via the miR-1258/RPN2 axis. SMAD2 knockdown upregulated RPN2 expression, while miR-1258 suppression or RPN2 upregulation significantly reversed these effects (Figure 7A-B). Moreover, rescue experiments showed that down-regulation of miR-1258 or the up-regulation of RPN2 partially abolished the circ_SMAD2 silencing- induced reduced cell proliferative and invasive abilities of CRC cells (Figure 7C-E).

### circ_SMAD2 modulates progression of CRC cells * in vivo*

To determine the effects of circ_SMAD2 on *in vivo* tumorigenesis, CRC cells were stably transfected with sh-NC, or sh-circ_SMAD2 and subcutaneously xenografted into nude mice. Compared to the sh-NC CRC cells, the sh-circ_SMAD2 CRC cells exhibited markedly small tumors (Figure 7F-G). Circ_SMAD2 knockdown significantly elevated miR-1258 levels and suppressed RPN2 expression (Figure 7H). These findings imply that circ_SMAD2 depletion impedes *in vivo* tumor growth. Therefore, circ_SMAD2 enhances CRC progression through the miR-1258/RPN2 axis (Figure 7I).

## Discussion

Recent developments in high-throughput sequencing technology have generated a lot of interest in the role of circRNAs in cancer 18. CircRNAs influence tumorigenesis by modulating various biological processes, including glycolysis, metastasis, and cell cycle 19. In hypoxic conditions, circ_MAT2B promotes liver cancer glycolysis and malignancy through the miR-338-3p-PKM2 axis 20. In addition, circRNA_0025202 regulates tamoxifen sensitivity in breast cancer by targeting the miR-182-5p-FOXO3a axis 21. The circ100284-miR-217-EZH2 axis influences arsenite-accelerated cell cycle in human keratinocytes during carcinogenesis 22. In this study, hsa_circ_0000847 (circ_SMAD2), a novel circRNA, was found to be markedly upregulated in CRC tissues and cell lines. Elevated circ_SMAD2 levels were correlated with advanced clinical features and poor CRC prognosis. Moreover, circ_SMAD2 knockdown significantly suppressed the *in vitro* proliferative and invasive rates of CRC cells and reduced the tumor growth *in vivo*, suggesting that circ_SMAD2 influences CRC progression.

Bioinformatic analyses identified 5 putative circ_SMAD2 miRNA targets and miR-1258 for further characterization. miR-1258 modulates multiple biological processes. The overexpression of miR-1258 suppresses osteosarcoma cell proliferation and G0/G1 by targeting AKT3 23. In addition, it suppresses lung cancer proliferation and induces apoptosis by modulating the GRB2/Ras/Erk pathway 24. Hsa_circ_0101432 enhances the proliferative and invasive ability of liver cancer by adsorbing mirR-1258 and miR-622 25. In this study, miR-1258 expression was suppressed in CRC cell lines and tissues. miR-1258 mimics inhibited the invasive and proliferative abilities of CRC cells. Therefore, miR-1258 confers anti-CRC effects. Circ_SMAD2 sponges miR-1258 in CRC.

Ribophorin II (RPN2) is an endoplasmic reticulum glycoprotein that modulates cell functions and signal transductions 26, 27. Elevated RPN2 levels have been documented to exhibit oncogenic functions in various cancers. Down-regulated RPN2 levels have been correlated with enhanced esophageal squamous cell carcinoma responses to docetaxel 28. RPN2 promotes liver cancer metastasis and reduces autophagy by regulating STAT3 and NF-κB signaling pathways 29. The *in vitro* invasive and proliferative abilities of colon carcinoma cells are hampered by the down-regulation of RPN2 30. However, the role of RPN2 in CRC progression has not been established. In this study, elevated RPN2 levels in CRC patients confirmed that RPN2 is a downstream target of miR-1258 in CRC cells. The overexpression of RPN2 reverses the effects of circ_SMAD2 silencing in CRC cells. This implies that circ_SMAD2/miR-1258/RPN2 is involved in CRC progression.

In conclusion, up-regulated circ_SMAD2 levels are associated with invasion and poor prognosis of CRC. In addition, circ_SMAD2 enhances malignancy by targeting miR-1258 to upregulate RPN2 expression in CRC. This is a potential therapeutic avenue against CRC.

## Figures and Tables

**Table 1:** The clinical features of cervical cancer patients

Characteristics		Number
Gender	Male	37
	Female	16
Age	<65	23
	≥65	30
Tumor size (cm)	<5	31
	≥5	22
Tumor site	Colon	25
	Rectum	28
Metastasis	No	38
	Yes	15
Tumor stage	I	8
	II	12
	III	18
	IV	15
